# Phylogeography of Chinese White Pine Beetle *Dendroctonus armandi* (Coleoptera: Curculionidae: Scolytinae) in China

**DOI:** 10.3390/genes17030292

**Published:** 2026-02-28

**Authors:** Hang Ning, Ruixiong Deng, Kaitong Xiao, Beibei Huang, Yu Cao, Qiang Wu

**Affiliations:** 1 Hubei Key Laboratory of Biological Resources Protection and Utilization, Hubei Minzu University, Enshi 445000, China; marsyu0722@163.com (R.D.); 13402709191@163.com (K.X.); hbei0825@163.com (B.H.); zhedaotinihui@163.com (Y.C.); lxwq20030226@163.com (Q.W.); 2 College of Forestry and Horticulture, Hubei Minzu University, Enshi 445000, China

**Keywords:** *Dendroctonus armandi*, mtDNA, divergence time, demographic expansion, phylogeographic pattern

## Abstract

Background: *Dendroctonus armandi*, an oligophagous beetle primarily infesting *Pinus armandii*, is geographically restricted and persistent in central China, causing significant ecological and economic losses. However, the intrinsic factors driving its continuous occurrence remain unclear. We examined the genetic variation patterns across the species’ range to explore its phylogeographic structure. Methods: We analyzed mitochondrial DNA sequence (mtDNA) data to assess population genetic structure and estimate the divergence times of distinct lineages. Results: Phylogenetic analysis identified four haplogroups corresponding to the Minshan (MSM), Qinling (QLM), Micang (MCM), and Ta-pa (TPM) Mountains. Demographic analyses revealed that QLM and TPM haplogroups have undergone population expansion events. Divergence time estimates indicated four lineages diverged during the Late Pleistocene. Notably, *D. armandi* may have followed two horizontal and one vertical independent colonization routes. The first route extended from MSM into QLM and then spread eastward along the QLM; the second route progressed from MSM into MCM and continued eastward into TPM; and the third route migrated southward from QLM into TPM. Conclusions: Climate oscillations, geographical isolation, and the patchy distribution of host trees collectively shaped the phylogeographic patterns of *D. armandi*. These findings elucidate the evolution and adaptability of *D. armandi* in mountainous environments.

## 1. Introduction

Over the past three million years, global climate has fluctuated dramatically. Alternating glacial and interglacial cycles during the Pleistocene epoch of the Quaternary period have crucially influenced species distributions and population differentiation [1,2]. Past Quaternary glacial–interglacial cycles have been suggested to shape the contemporary distribution of Northern Hemisphere species [1]. However, the effects of these drastic climate shifts varied across different regions. During glacial periods in North America, refugia included the Rocky Mountains, high-latitude Arctic regions, the Bering area, and the southern Appalachian Mountains. Previous studies have confirmed that these regions served as refugia for both plants and animals [3]. In Europe, the Italian, Iberian, and Balkan Peninsulas, located south of glaciers, functioned as glacial refugia for numerous species. The unique geography of these peninsulas limited gene flow among populations, giving rise to distinct evolutionary patterns and genetic lineages [4]. Although the Asian and European continents share similar latitudes in the Northern Hemisphere, species distributed across Eurasia underwent divergent evolutionary histories during the Quaternary glacial periods, with varying effects from the immense climatic shifts caused by glacial events [2,5]. During the Pleistocene of the Quaternary glacial period, the Last Glacial Maximum (LGM) had a pronounced effect on Eurasia, especially on species distributed in Europe, which experienced large-scale population expansions after the LGM [6]. For species distributed in Asia, population expansion mainly occurred before the LGM, whereas in some regions (such as the eastern Himalayas), populations remained relatively stable [7,8,9].

Compared with other countries in Eurasia, the geological structure of China is more complex, with local characteristics attributed to the influence of the Quaternary Ice Age. China has experienced several glacial cycles [10]. The main Quaternary glacial periods with far-reaching influence include the LGM, the penultimate glacial period, and the antepenultimate glacial period [11,12]. Among these, the LGM had a profound effect on biological groups in China. Notably, population expansion occurred after the last glacial period, which has become a widely existing evolutionary pattern for many biological groups in China [13,14,15,16]. The population expansion patterns before and after the LGM have become fundamental strategies for some birds and insects to cope with climate change [9,17]. Beyond the LGM, the penultimate glacial period also influenced species differentiation in China, particularly among some plants, birds, and insects, leading to relatively pronounced population differentiation [8,14,18]. The Qinling and Ta-pa Mountains serve as the boundary between the Palaearctic and Oriental regions, as well as the climatic divide between temperate and subtropical zones [19]. Species distributed across these mountains exhibit distinct phylogeographic patterns: north–south (longitudinal) and east–west (latitudinal) genetic differentiation [13,20,21,22,23,24]. The northern and southern Qinling Mountains exhibit significant differences in habitat, climate, and species diversity. This region serves as a transitional zone between temperate deciduous forests and subtropical evergreen forests, functioning as a center of diversity and origin for certain biological groups [25,26]. The Ta-pa Mountains, situated south of the Qinling, run roughly parallel to them and are separated by the Hanzhong Plain and Han River. Both the Qinling and Ta-pa Mountains exhibit high biodiversity and served as common glacial refugia [14,20]. Climatic oscillations and the isolation imposed by mountains and rivers may have led to genetic variation and differentiation among populations of certain species in this region.

Bark beetles comprise over 6000 species within approximately 225 genera, specializing in the exploitation of mainly hardwood trees and conifers [27]. Currently, researchers have used phylogeography to explore the evolution of bark beetle genera such as Dendroctonus, Ips, Tomicus, and Pityogenes. Among them, the genus Dendroctonus has over 20 species worldwide, most of which occur in North and Central America, where they are considered major pests of conifer forests [28]. In 2007, Mock et al. analyzed North American *Dendroctonus ponderosae* populations across their range using both mitochondrial DNA (mtDNA) COI and COII, and AFLP. The results revealed a significant genetic structure among populations that followed a broad isolation-by-distance pattern [29]. In 2007, Maroja et al. analyzed *D. rufipennis* populations using mtDNA COI and nine microsatellites. The results revealed the occurrence of three genetically distinct haplotype groups, suggesting the existence of major population groupings associated with different geographical regions. Additionally, the deep divergence between the groups was explained by the combined effect of several glacial cycles, when beetles spread from at least three refugia [30]. A study based on mtDNA COI conducted by Anducho-Reyes et al. determined the phylogeographic structure of *D. mexicanus* populations collected in the mountains of Mexico and Guatemala. The results revealed that *D. mexicanus* population differentiation was first determined by the conformation of the Mexican mountain systems and then by the dispersal ability of the beetle. *D. mexicanus* experienced a rapid population expansion during its dispersal across mountain systems within its current range. The emergence of geographical barriers during the Pleistocene promoted isolation events that facilitated *D. mexicanus* populations to follow divergent evolutionary routes [31].

*Dendroctonus armandi* (Coleoptera: Curculionidae: Scolytinae) is a small endophytic pest that completes its life cycle under the bark of its host, *Pinus armandii* [32]. As a pioneer species, it unites with the blue-stain fungus *Leptographium qinlingensis* to invade healthy hosts for over 30 years, and triggers secondary bark beetles to attack infected or withered host trees [32,33]. Females are always the first to bore through the bark of the host and then attract males with aggregation pheromones for colonization and reproduction [32]. Voltinism varies with elevation in the Qinling and Ta-pa Mountains. Typically, two generations per year occur at elevations lower than 1700 m, three generations within 2 years between 1700 and 2150 m, and one generation per year above 2150 m [19]. Many studies have shown that the evolutionary histories of species from the Qinling and Ta-pa Mountains were shaped by Pleistocene glacial processes [14]. These non-glaciated regions may represent a variety of refugia for species in the presence of climatic changes during the Pleistocene. However, no equivalent phylogeographic hypothesis has been proposed regarding the distribution of *D. armandi* populations. Based on the findings of common genetic patterns of species distributed across their geographical ranges, we hypothesized that a similar relationship may exist among *D. armandi* populations.

In the present study, we used mtDNA COI to examine the genetic structure of *D. armandi* populations, estimate the divergence times of distinct lineages, and determine the colonization and migration routes of *D. armandi* populations. Finally, we aimed to propose a basic framework for the phylogeography of *D. armandi* based on these findings.

## 2. Materials and Methods

### 2.1. Sample Collection and DNA Amplification

**Figure 1:**
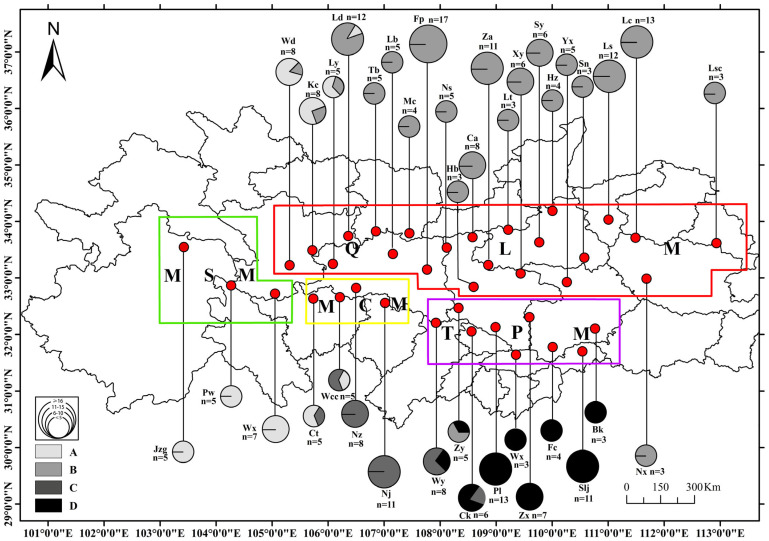
Sample distribution and haplotype frequency of the COI mtDNA of 38 *D. armandi* populations. Number of individuals for each geographical area is given by n. Pie graphs represent the frequency of COI haplotypes belonging to each haplogroup: (A) MSM = Minshan Mountains; (B) QLM = Qinling Mountains; (C) MCM = Micang Mountains; (D) TPM = Ta-pa Mountains. These haplogroups reflect phylogenetic results from Figure 2.

We collected 255 *D. armandi* adults from 38 localities spanning its entire geographic distribution range (Table 1; Figure 1). All individuals were collected from infested trees in 2024–2025, preserved in 95% ethanol, and stored at −80 °C until DNA extraction. DNA extraction was performed using a DNeasy Tissue Kit (Qiagen, Valencia, CA, USA) according to the manufacturer’s protocol. Amplification and sequencing of mtDNA COI were conducted using the primers C1-J-2441 and T12-N-3014, as previously described by Ruiz et al. [34]. Amplicons were purified using GFX polymerase chain reaction DNA and Gel Band Purification Kit (Amersham Bio-sciences, Buckinghamshire, UK), and sequenced with an ABI Prism 3100 Genetic Analyzer (Applied Biosystems, Foster City, CA, USA). We did not observe polymerase chain reaction ghost bands nor find a significant proportion of sequence ambiguities, which suggests that no nuclear pseudogenes of mtDNA (Numts) were amplified [35]. All obtained nucleotide sequences were compared, edited manually, and aligned using the Sequencher^®^ 4.7 software. The mitochondrial sequences used in this study have been deposited in GenBank under accession no. PX924952 to PX924998. All sequences obtained correspond to positions between 2428 and 2977 in the *Drosophila yakuba* mitochondrial genome (accession X03240). This particular region was chosen because previous sequence analyses revealed that nucleotide divergence is appropriate to assess population differences in *D. armandi*, which has also been observed in other Dendroctonus species [31].

### 2.2. Phylogenetic Reconstruction

To reconstruct the phylogenetic relationships among the mtDNA haplotypes, the maximum-likelihood (ML) algorithm implemented in the aLRT-PHYML program was used [36]. Before the ML analysis, an appropriate model of DNA evolution and model parameters were determined using both the Akaike Information Criterion and hierarchical likelihood ratio tests. Tree topology rather than branch lengths was optimized in the aLRT-PHYML analyses. To estimate the reliability of each node, the approximate likelihood ratio test with the Shimodaira–Hasegawa-like procedure option was used. The sister species, *Dendroctonus simplex* LeConte, was used as the outgroup (accession AF067985). The main clades resulting from the phylogenetic reconstruction were used as natural groups (haplogroups) to analyze the demographic history and divergence time between *D. armandi* populations. The number of segregating sites, haplotypes, haplotype diversity, and the average number of nucleotide differences for each haplogroup alone and for all haplogroups combined were estimated using DnaSP v6.12.03 [37]. To identify departures from the mutation–drift equilibrium that could indicate natural selection, the Tajima’s *D* statistic, Fu and Li’s *D*, and Fu’s *F_s_* were also estimated for the identified haplogroups. These tests were implemented using both DnaSP and Arlequin v3.5.2.2 [38].

### 2.3. Demographic History

**Figure 2:**
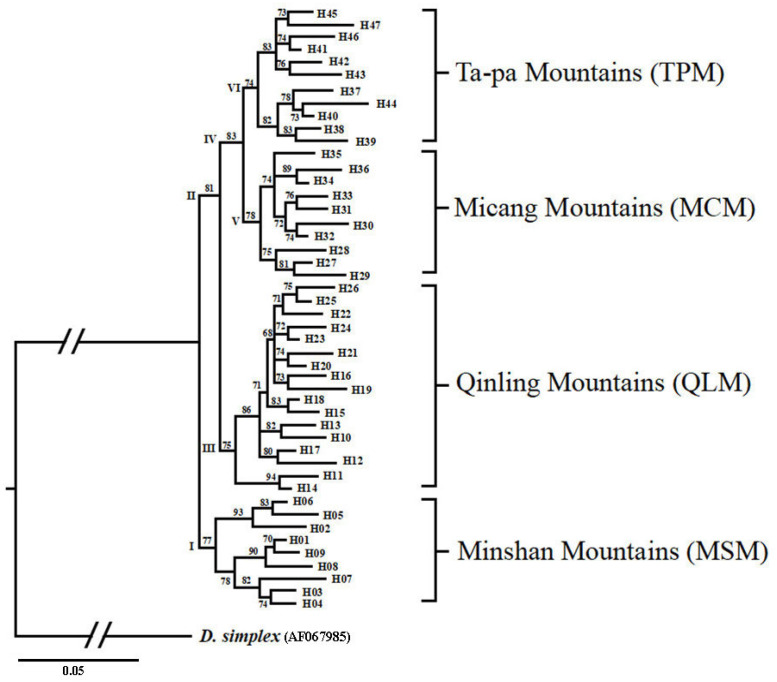
ML tree reconstruction based on 596 bp of the mtDNA COI of the *D. armandi*.

Two different approaches were adopted to analyze the temporal changes in effective population size, both of which were designed to test the major haplogroups identified in the molecular phylogenetic analyses (Minshan Mountains = MSM, Qinling Mountains = QLM, Micang Mountains = MCM, and Ta-pa Mountains = TPM; Figure 2). These approaches were as follows: (1) neutrality tests against population growth and (2) the distribution of pairwise differences [36]. Notably, Tajima’s *D* statistic, despite its original purpose of testing selective neutrality, can also be applied to infer the demographic history of a population. In demographically stable populations at equilibrium, the estimated value of *θ* (*θ* = 2N_e_*μ*) is identical whether derived from the number of segregating sites (*θ_S_*) or pairwise nucleotide differences (*θ_π_*). Because *θ_π_* assesses the frequency of the mutant alleles, it is more sensitive to recent changes in effective population size. Therefore, negative values of Tajima’s *D* statistic values can reveal recent demographic expansions. The Fu’s *F_s_* and the Ramos-Onsins and Rozas’s *R*_2_ neutrality tests were also used, as both have been demonstrated to have superior statistical power regardless of sample size or number of mutations and for reasonable population growth parameters [39]. To test for significance, 10,000 random data simulations were generated under the hypothesis of selective neutrality and population equilibrium. The three tests were implemented using either DnaSP or Arlequin.

Subsequently, a mismatched distribution was employed to test for sudden population expansion. In this approach, the distribution of pairwise sequence differences is expected to be multimodal when haplotypes are drawn from populations at demographic equilibrium but unimodal when haplotypes are drawn from populations that have undergone sudden demographic expansion. To reflect sudden population growth from N_0_ to N_1_ at *t* preceding generations, the approach implemented in Arlequin assumes a stepwise expansion model. Following the methodology of Schneider and Excoffier, three demographic parameters were estimated: *θ*_0_ = 2*μ**N***_0_, *θ*_1_ = 2*μ**N***_1_, and τ = 2*ut*, with *u* denoting the mutation rate across the whole gene region [38]. The goodness-of-fit between the observed data and the simulated expansion model was tested using the sum of squared deviations. Additionally, we calculated the raggedness index (*rg*) of Harpending’s. When the validity of the model was confirmed, we further investigated the time since the putative expansion event from τ was further investigated, using a generation time of this insect as 1.5 years (average), and a divergence rate of 1.1% per million years [40]. For all these parameters, confidence intervals (95%) were obtained using 10,000 randomizations of the data.

### 2.4. Divergence Time Between D. armandi Populations

To estimate divergence times among the haplogroups of *D. armandi* populations, the MDIV v1.0, which enables joint estimates of divergence times and migration rates of two populations, was used [41]. MDIV estimates *θ* (=2N_e_*μ*) and *T* (the divergence time between populations, where one time unit = N_e_ generations). The program was first run using default search settings and priors. Subsequently, the prior maximum value of M_max_ (M = scaled migration rate) and T_max_ (T = scaled divergence time) values were set to 10 and 5, respectively, as in previous runs, and the MDIV produced consistent and well-behaved posterior distributions. The integrated likelihood function surfaces were obtained using the finite-sites model HKY, with 5,000,000 generations of the Metropolis-Hastings Markov chain Monte Carlo and a 1,000,000-generation burn-in time to explore the solution space. This was repeated three times to ensure convergence upon the same posterior distributions for each parameter estimate. The modes of posterior distribution for both θ and *T* were used to estimate divergence times between *D. armandi* haplogroups, according to the formula: *T*_pop_ = [(*t*_pop_θ)/2*L*] 1/*μ*, where *T*_pop_ is the population divergence time, *L* is the sequence length (596 bp), and *μ* is the mutation rate per site per generation. In this program, the commonly cited 1.1% per million years per lineage for arthropod mtDNA was also employed [40].

## 3. Results

### 3.1. Molecular Genealogy of the D. armandi Haplotypes

We sequenced 596 nucleotides of mitochondrial COI and identified 142 variable sites, leading to the identification of a total of 47 different haplotypes. ML analysis performed with the 47 haplotypes resulted in a single tree (Figure 2). Clade I was monophyletic and included nine haplotypes from three locations (JZG, PW and WX) in the MSM. Clade II had two main subclades: one subline from the QLM (Clade III) occupied 17 haplotypes at 22 geographical locations along the mountain range (Wd, Kc, Ld, Ly, Lb, Fp, Ns, Hb, Xy, Tb, Mc, Ca, Lt, Hz, Za, Sy, Sn, Yx, Ls, Lc, Nx and Lsc). Among them, Foping County, located in the middle of the QLM, had the highest number of haplotypes (seven). Clade IV also had two subclades: one from MCM (Clade V) and the other from TPM (Clade VI). Clade V contained 10 haplotypes from four geographical locations (Ct, Wcc, Nz and Nj) along the mountain range. Clade VI included 11 haplotypes from nine geographical locations (Zy, Wy, Ck, Pl, Wx, Zx, Fc, Bk, and Slj). These clades had strong nodal support values, 99% of them have exceeded 70. However, the scores of these branch nodes ranged between 70 and 82. Despite their seemingly low statistical branch supports, these clades are considered reliable groups because the probability of an inferred branch is directly proportional to the cutoff probability, and the aLRT test tends to perform as well as other branch tests (based on nonparametric ML bootstrap supports) or better (Bayesian posterior probabilities), with a cutoff probability between 60 and 80 [42]. Overall, *D. armandi* consisted of four haplogroups, corresponding to MSM, QLM, MCM, and TPM.

**Figure 3:**
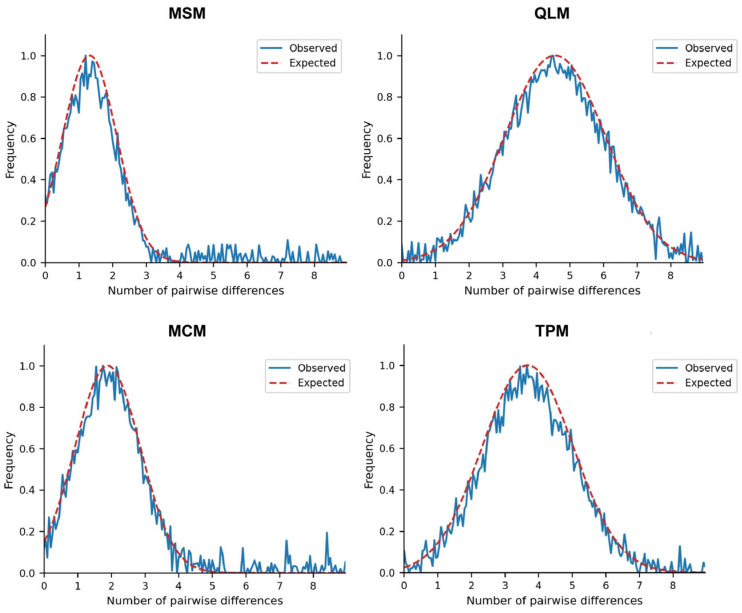
Demographic history inferred by mismatch distributions of numbers of pairs of nucleotide differences among individuals within each of the four haplogroups (Observed distributions: blue lines; expected distributions: red line).

All haplogroups exhibited high haplotype diversity (*Hd* = 0.934–0.952), with the number of variable sites (*S*) ranging from 23 to 57 and haplotypes (*Nhap*) ranging from 9 to 17. The QLM haplogroup exhibited the highest genetic diversity (*S* = 57, *Nhap* = 17, *π* = 0.047, *k* = 27.8), accompanied by a significantly negative Fu and Li’s *D* (−3.892) (Table 2). Combined with its unimodal mismatch distribution, this confirmed recent sudden population expansion (Figure 3). In contrast, MSM and MCM had relatively low genetic diversity (*S* = 23/27, *π* = 0.019/0.022) and weakly negative Fu and Li’s *D* values (−1.256/−1.568), indicating stable evolutionary histories without obvious population expansion. In addition, MSM and MCM exhibited weakly negative nonsignificant Tajima’s *D* and Fu’s *F_s_* values, reflecting no departures from the mutation–drift equilibrium (Table 2 and Table 3). The TPM haplogroup displayed intermediate genetic diversity, consistent with its moderate population expansion signal (Figure 3).

### 3.2. Divergence Time

Population divergence times among the haplogroups ranged from 0.265 to 0.492 Myr, with the deepest divergence observed between QLM and TPM (0.492 Myr) and the shallowest between MSM and MCM (0.265 Myr) (Table 4). The *T*_MRCA_ values (0.289–0.518 Myr) were consistently slightly higher than the corresponding *T*_pop_ values for all haplogroup pairs, conforming to the expected genetic pattern of ancestral coalescence preceding population divergence. Estimates of *θ* (2.23–3.68) and effective female population size (N_e_♀: 2401–4521) were highest for the QLM–TPM pair, reflecting greater genetic differentiation and larger historical population sizes in these expanding haplogroups. These divergence time estimates, all falling within the Late Pleistocene, align with the demographic expansion signals detected in QLM and TPM populations, including significantly negative Tajima’s *D* and Fu’s *F_s_* values, low *SSD* and *rg*, unimodal mismatch distributions, *θ*_0_ ≈ 0 and positive *τ* estimates. In contrast, MSM and MCM showed stable population dynamics with non-significant neutrality tests, higher *SSD* and *rg* values, and flat mismatch distributions, consistent with their relatively shallow divergence from the other haplogroups (0.265–0.385 Myr).

## 4. Discussion

Correctly evaluating the population differentiation and phylogenetic relationships among populations of *D. armandi* has been a challenging task; however, the intraspecific relationships for different lineages of *D. armandi* are clear. Our molecular data analysis suggested that *D. armandi* comprises four main lineages with almost unique sets of haplotypes and distinct geographic distributions in the Minshan, Qinling, Micang, and Ta-pa Mountains. Previous research on potential Quaternary glacial refugia in China has identified numerous potential refugia, including the Hengduan, Qinling, and Ta-pa Mountains [16]. In the present study, all populations of *D. armandi* were also confined to these geographical areas and were distributed from west to east along the Min and Qin-ba Mountains. Influenced by the uplift of the Qinghai–Tibet Plateau, the topography and geomorphology of this region became highly complex, particularly in terms of climate and geology. Numerous studies have confirmed that geographic isolation in this area has segregated populations, impeded gene flow, and generated genetic differentiation, which are key constraints on the unique geographic distribution of *D. armandi*. In summary, our findings contribute to a better understanding of the geographical distribution patterns and evolutionary history of *D. armandi*.

Previous studies have found that *D. armandi* inhabits the southern slopes of valleys or canyons at altitudes of 1300–2400 m [19]. Geographic barriers may have influenced the phylogeographic structure of *D. armandi.* For species with limited migratory capacity and weak flight abilities, such geographic barriers drive the formation of new species (e.g., allopatric speciation) among isolated populations [16,43]. The accumulation of independent genetic differences is expected in populations that evolved in allopatry, and higher genetic variability is also expected in ancient populations compared with recent populations, because the latter are frequently a subset arising from the original gene pool. Such differences may be used as genetic markers to trace expansion routes [6]. During the Pleistocene, the periodic expansion and contraction of glaciers led species to survive in refugia during the ice age and expand to other areas afterward. The haplotype and nucleotide diversity results showed that MSM, QLM, MCM, and TPM were the ancient refugia for *D. armandi* populations. The MSM (belonging to the Hengduan Mountains) is located in the northeast of the Qinghai–Tibet Plateau, at the intersection of northern Sichuan Province and southern Gansu Province. This bark beetle may have survived during the Ice Age and subsequently expanded into surrounding areas. The haplotype and nucleotide diversity in the QLM were higher than those in the MCM. Therefore, we inferred that *D. armandi* first spread and colonized the QLM from west to east. In addition, the bark beetle may have spread from MSM to MCM via another west-to-east route. However, the haplotype and nucleotide diversity in the TPM (up to Shennongjia) were higher than those in the MCM. Similar results in other North American and European bark beetles revealed that many range expansion events were linked to postglacial colonization from Pleistocene refugia [29,30,31].

Our results confirmed the existence of four well-supported haplogroups (MSM, QLM, MCM, and TPM) of *D. armandi* based on mitochondrial COI gene sequences, with a clearly resolved phylogenetic topology and distinct genetic structure among geographically differentiated lineages. The inclusion of a comprehensive set of sampling sites covering the entire distribution range of *D. armandi* and the identification of 47 haplotypes provided a more clearly resolved phylogenetic reconstruction. Clades I and II had a basal position and similar branch lengths, indicating that the initial divergence of *D. armandi* haplogroups may have occurred in close temporal proximity. The mummified *Pinus* L. woods from the Upper Pleistocene of the Maoming Basin, Guangdong Province, South China, indicate that the cold-resistant plant *P. armandii* expanded to the low-latitude areas of South China during the Last Glacial Period [44]. Subsequently, *P. armandii* contracted again during the interglacial period. Meanwhile, *P. armandii* distributed in the Qinling and Ta-pa Mountains and in the Yunnan–Guizhou Plateau of central and southwest China were affected by the cooling effect of the ice age, allowing the cool-loving *P. armandii* to spread to lower altitude areas. After the end of the ice age, the climate warmed, and its distribution gradually retreated to suitable high-altitude habitats [44,45]. The main reason for this phenomenon may be that temperature changes during the last ice age affected the seed reproduction and spread of *P. armandii*. As a large-seeded pine species, it has a short dispersal distance, limited to the area around parent trees, and mainly relies on animal-mediated dispersal, all of which adversely affect its spread [45]. Coupled with geographical barriers such as mountains and rivers, communication between populations is almost completely blocked, making short-distance dispersal dominant within a local geographic range. The recovery of *P. armandii* populations through natural regeneration is very difficult due to multiple limitations on migration caused by phenotypic plasticity, seed dispersal, and fecundity, eventually resulting in habitat fragmentation and a patchy distribution [46,47]. Ancient populations of *D. armandi* survived in isolated refugia during glacial periods, driving the initial divergence of the four haplogroups. Notably, the topological structure also showed that the relationship between the populations in TPM and MCM was closer than that between QLM and TPM. This pattern may reflect the combined effects of postglacial range expansion, limited gene flow among refugial populations, and host plant specialization, which have collectively shaped the current genetic structure of *D. armandi*.

Generally, nucleotide variation at silent sites is closer to the neutral evolutionary pattern and thus can more accurately reflect the degree of nucleotide variation in a species [48]. Although many of the previously mentioned results can be used as evidence of historical demographic changes (shallow star-like phylogeny, high haplotype and nucleotide diversity, and low frequency of older, more divergent lineages), we specifically tested the hypothesized demographic expansion using two different approaches: neutrality tests against population growth and mismatch distribution. The neutrality tests suggested that populations from QLM and TPM experienced demographic expansion events. Combined with the indicators in Table 3, the results from all approaches strongly support the hypothesized demographic expansion of the QLM and TPM haplogroups. In contrast, MSM and MCM showed stable population sizes with no evidence of recent demographic expansion. The estimated times of the putative expansion events from all mountain haplogroups were in the late Pleistocene, probably after a period of strong bottlenecks due to past glaciations. Our results also agree with those of other studies performed with species of similar distributions [29,30,31]. Nonetheless, caution must be exercised when interpreting these results, as a single-locus analysis cannot exclude different models of selection rather than demographic expansion.

The continuous uplift of the Qinghai–Tibet Plateau is key to establishing climate patterns in East Asia [49]. Since the Quaternary period, the surrounding mountains have been influenced by external forces such as glaciers and water erosion, ultimately forming the current topography [50]. Undoubtedly, climate fluctuations and geographical isolation may have been the original forces behind the differentiation of *D. armandi*. Estimates of the divergence time between QLM and TPM haplogroups yielded 0.492 Myr during the Late Pleistocene, in contrast to their differentiation times, which were earlier. One possible explanation is that the *D. armandi* lineages within these two haplogroups experienced significant divergence, but with some intermittent contact over time and continuous eastward expansion into Shennongjia long before the last glacial maxima. Lineages in TPM have experienced differences in host availability as a direct consequence of climate change. In this scenario, the eastern range of *P. armandii* in TPM may have provided multiple refugia, whereas glaciers covered most of its western range. These results align with the evolutionary history of the host *P. armandii*, suggesting that warmer interglacial climates pushed conifers eastward from QLM to TPM. However, environmental changes reduced the population size of *P. armandii* and forced fragmentation of the distribution range in QLM and TPM. These events may have led to the isolation of populations from QLM and MCM and promoted the divergence of other lineages. Ultimately, a branch of *D. armandi* was formed in TPM. In short, many factors affect the distribution pattern (genetic structure) of species, including the distribution of sampled individuals, sampling principles, the evolutionary rate of the studied gene fragments, and historical dynamic changes in natural selection or populations. Additionally, population isolation or distribution fragmentation caused by environmental and human interference must also be considered. Beyond these, long-term climatic oscillations, geological events, topographic heterogeneity, and habitat changes can promote long-term population isolation and restricted gene flow [51]. Meanwhile, intensified anthropogenic disturbances—such as deforestation, agricultural expansion, urbanization, road construction, overexploitation, and habitat loss and fragmentation—further disrupt population connectivity, reduce effective population size, and accelerate genetic differentiation [52]. Together, these natural and human-induced processes collectively shape the contemporary genetic architecture and distributional patterns of species. Therefore, future research should jointly explore these factors to better understand the formation mechanisms of the geographical distribution pattern of *D. armandi*.

## 5. Conclusions

The mtDNA analysis of 255 individuals from 38 populations identified 45 geographically structured haplotypes, which formed four haplogroups corresponding to the MSM, QLM, MCM, and TPM. Among four haplogroups, the QLM and TPM haplogroups underwent demographic expansion, with all inter-haplogroup divergence times dating from the late Pleistocene. *D. armandi* exhibited three independent colonization routes: two latitudinal (MSM→QLM; MSM→MCM→TPM) and one longitudinal (QLM→TPM). Apart from climate fluctuations and geographical isolation, the patchy distribution of its host *P. armandii* further reinforced this phylogeographic pattern. These results enhance understanding of evolution and adaptation for *D. armandi* in mountainous systems.

## Figures and Tables

**Table 1:** Samples of *D. armandi* used for molecular analyses in this study.

Mountain System and Collecting Locality	Abbr.	Latitude/Longitude	No. Samples	Haplotype
**Minshan Mountains**	**MSM**			
Jiuzhaigou	Jzg	103°54′ E/33°10′ N	5	H01(2), H03(2), H06
Pingwu	Pw	104°18′ E/33°01′ N	5	H01, H02(2), H04(2)
Wen	Wc	105°7′ E/32°39′ N	7	H02(2), H05(2), H07, H08, H09
**Qinling Mountains**	**QLM**			
Wudu	Wd	105°23′ E/33°13′ N	8	H02(3), H06(2), H07(3), H14
Kang	Kc	105°44′ E/33°25′ N	8	H03, H08, H11, H12(3), H17(2)
Liangdang	Ld	106°27′ E/33°41′ N	12	H05, H10(3), H13(2), H15(4), H17, H18, H19
Lueyang	Ly	106°8′ E/33°15′ N	5	H03(2), H10, H11(2)
Liuba	Lb	107°16′ E/33°46′ N	5	H10, H15, H17, H19(2)
Foping	Fp	108°2′ E/33°17′ N	17	H13(3), H16, H17(4), H19(2), H20(3), H21(2), H22(2)
Ningshan	Ns	108°12′ E/33°26′ N	5	H19(2), H23, H24(2)
Hanbin	Hb	108°53′ E/32°57′ N	3	H17(2), H22
Xunyang	Xy	109°30′ E/33°4′ N	6	H18(2), H20, H21, H22, H24
Taibai	Tb	106°26′ E/33°59′ N	5	H17, H18(2), H20(2)
Mei	Mc	107°46′ E/34°2′ N	4	H10(3), H20
Changan	Ca	109°5′ E/33°57′ N	8	H17, H19(3), H22, H23(3)
Lantian	Lt	109°22′ E/33°50′ N	3	H19, H25(2)
Huazhou	Hz	110°4′ E/34°29′ N	4	H22, H24, H25(2)
Zhenan	Za	108°56′ E/33°11′ N	11	H20, H21(3), H22(2), H23(2), H25(3)
Shanyang	Sy	109°51′ E/33°38′ N	6	H21(2), H23(2), H25, H26
Shangnan	Sn	110°35′ E/33°18′ N	3	H19, H24, H25
Yunxi	Yx	110°21′ E/32°57′ N	5	H17, H19(2), H23, H25
Lushi	Ls	111°7′ E/34°0′ N	12	H19(3), H20(4), H22(3), H23(2)
Luanchuan	Lc	111°26′ E/33°46′ N	13	H17(3), H19(5), H20, H23(2), H25(2)
Neixiang	Nx	111°45′ E/33°3′ N	3	H23, H25, H26
Lushan	Lsc	112°48′ E/33°37′ N	3	H23, H25(2)
**Micang Mountains**	**MCM**			
Chaotian	Ct	105°49′ E/32°38′ N	5	H05(3), H28, H29
Wangcang	Wcc	106°21′ E/32°39′ N	5	H08(2), H27, H28, H32
Nanzheng	Nz	106°33′ E/32°44′ N	8	H26(2), H28(3), H29, H30, H31
Nanjiang	Nj	107°1′ E/32°38′ N	11	H29(3), H30, H33(3), H34(2), H35, H36
Ta-pa Mountains *	**TPM**			
Ziyang	Zy	108°24′ E/32°24′ N	5	H17(2), H23, H39(2)
Wanyuan	Wy	107°57′ E/32°8′ N	8	H30(3), H33, H34(2), H38(2)
Chengkou	Ck	108°39′ E/32°2′ N	6	H30, H32, H33(2), H36, H40
Pingli	Pl	109°7′ E/32°8′ N	13	H37(3), H38, H39(4), H40, H41(3), H43
Wuxi	Wx	109°26′ E/31°37′ N	3	H39, H40, H41
Zhuxi	Zx	109°36′ E/32°14′ N	7	H40(4), H41(2), H44
Fang	Fc	110°4′ E/31°48′ N	4	H42, H43(3)
Baokang	Bk	111°7′ E/31°58′ N	3	H46, H47(2)
Shenlongjia	Slj	110°39′ E/31°40′ N	11	H38, H40(2), H41, H42(3), H45, H46, H47(2)

Numbers in parentheses correspond to haplotype frequencies; * Ta-pa Mountains in this study refers to the narrow sense of Ta-pa Mountains, excluding Micang Mountains.

**Table 2:** Nucleotide polymorphism and results of neutrality test for the mtDNA COI of *D. armandi*.

Haplogroup	*S*	*Nhap*	*Hd*	*π* (SD)	*k*	Fu and Li’s *D*
MSM	23	9	0.943	0.019 ± 0.003	11.2	−1.256
QLM	57	17	0.952	0.047 ± 0.006	27.8	−3.892
MCM	27	10	0.934	0.022 ± 0.004	13.1	−1.568
TPM	35	11	0.949	0.029 ± 0.005	17.0	−2.684
ALL	142	47	0.961	0.059 ± 0.007	35.3	−4.987

*S*, no. of segregating sites; *Nhap*, no. of haplotypes; *Hd*, haplotype diversity; *π*, nucleotide diversity; *k*, average number of nucleotide differences; Fu and Li’s *D*, statistic of Fu and Li’s *D* test.

**Table 3:** Statistical tests for selective neutrality and population expansion indices based on mtDNA COI of *D. armandi*.

Haplogroup	MSM	QLM	MCM	TPM	ALL
No. of sequences	17	149	29	60	255
Tajima’s *D*	−0.918	−3.025 ***	−1.075	−1.928 *	−3.268 ***
Fu’s *F_s_*	−2.286	−23.896 ***	−3.108	−9.352 **	−30.569 ***
R_2_	0.130	0.052	0.112	0.075	0.042
SSD	0.008	0.002	0.007	0.004	0.001
*rg*	0.063	0.026	0.057	0.040	0.020
*τ* (95%CI)	1.32 (0~3.78)	4.56 (1.82~6.95)	1.89 (0~4.32)	3.68 (1.05~6.12)	5.12 (2.18~7.96)
*θ*_0_ (95%CI)	0.05 (0~2.29)	0 (0~1.05)	0.04 (0~2.58)	0 (0~1.82)	0 (0~0.92)
*θ*_1_ (95%CI)	3.45 (0.92~7.73)	16.98 (7.25~25.63)	4.76 (1.28~9.85)	10.56 (3.89~17.25)	19.89 (8.65~30.52)

Tajima’s *D*, Tajima’s *D* statistic; Fu’s *F_s_*, statistic of Fu’s *F_s_* test; R_2_, Ramos-Onsins and Rozas’s R_2_ statistics; SSD, sum of squared deviations; *rg*, Harpending’s raggedness index. Demographic parameters: *θ*_0_ = 2*μ*N_0_; *θ*_1_ = 2*μ*N_1_; and *τ* = 2*ut*; CI = confidence interval. *, *p* < 0.05; **, *p* < 0.01; ***, *p* < 0.001.

**Table 4:** Results of the migration/isolation model and divergence time among haplogroups of *D. armandi*, obtained using the MDIV software.

Haplogroup 1	Haplogroup 2	*θ*	N_e_♀	*T*	*T*_MRCA_	*T_pop_* (Myr)
MSM	QLM	2.86	3528	8.95	0.412	0.385
MSM	MCM	1.95	2401	6.28	0.289	0.265
MSM	TPM	2.42	2976	7.63	0.351	0.328
QLM	MCM	3.05	3756	9.58	0.443	0.418
QLM	TPM	3.68	4521	11.25	0.518	0.492
MCM	TPM	2.23	2745	6.95	0.319	0.296

N_e_♀, female effective population size; *T*, scaled divergence time; *T*_MRCA_, time to the most recent common ancestor (measured in units of N_e_♀ generations); *T_pop_*, population divergence time among indicated haplogroups.

## Data Availability

The original contributions presented in this study are included in the article. Further inquiries can be directed to the corresponding author.

## Abbreviations

The following abbreviations are used in this manuscript:

MSM	Minshan Mountains
QLM	Qinling Mountains
MCM	Micang Mountains
TPM	Ta-pa Mountains
COI	Mitochondrial DNA Cytochrome c Oxidase Subunit I
LGM	Last Glacial Maximum
RAPD	Random Amplified Polymorphic DNA
ML	Maximum Likelihood
